# Oral Health-Related Quality of Life in Patients With Chronic Respiratory Diseases—Results of a Systematic Review

**DOI:** 10.3389/fmed.2021.757739

**Published:** 2022-01-12

**Authors:** Simin Li, Wanchen Ning, Wei Wang, Dirk Ziebolz, Aneesha Acharya, Gerhard Schmalz, Jianjiang Zhao, Shaohong Huang, Hui Xiao

**Affiliations:** ^1^Stomatological Hospital, Southern Medical University, Guangzhou, China; ^2^Department of Cariology, Endodontology and Periodontology, University Leipzig, Leipzig, Germany; ^3^Dr. D. Y. Patil Dental College and Hospital, Dr. D. Y. Patil Vidyapeeth, Pune, India; ^4^Shenzhen Stomatological Hospital, Southern Medical University, Shenzhen, China

**Keywords:** oral health, oral health-related quality of life, respiratory disease, lung, COPD

## Abstract

**Background:** This systematic review evaluates the oral health-related quality of life (OHRQoL) of patients with chronic respiratory diseases.

**Methods:** A systematic literature search was performed based on the PubMed, Medline, Web of Science, and Scopus, using the search terms: “oral health-related quality of life” and “respiratory disease” or “lung” and “oral health-related quality of life.” Full-text articles published until June 30, 2021 and reporting any OHRQoL measurement in children or adults with a chronic respiratory disease or condition were included and analyzed qualitatively.

**Results:** A total of seven out of 44 studies were included, of which four studies examined adults and three studies investigated children. The respective diseases were chronic obstructive pulmonary disease (COPD) (*n* = 2), sleep apnea (*n* = 2), severe asthma (*n* = 1), cystic fibrosis (*n* = 1), and lung transplantation (*n* = 1). Four studies confirmed a worse OHRQoL in the respiratory diseased group compared to healthy controls. The overall OHRQoL was reduced in the included studies. Oral health, health-related quality of life, and disease-related parameters were rarely examined with regard to OHRQoL.

**Conclusion:** Patients with chronic respiratory diseases show a reduced OHRQoL. Oral health should be fostered in these individuals to support their OHRQoL.

## Background

Chronic diseases affecting the respiratory system cover a broad variety of different conditions; thereby, lifestyle or risk factor-associated, high prevalent diseases, such as chronic obstructive pulmonary disease (COPD), exist (1) alongside with very rare genetic diseases, such as cystic fibrosis (2). Other examples of respiratory diseases are asthma, which is a highly prevalent condition with primarily an autoimmune cause (3) or sleep apnea or sleep-disordered breathing as a complex and multifactorial condition (4). Especially, if breathlessness occurs, chronic respiratory diseases can be related to a high morbidity and a negative impact on the quality of life of affected patients (5). Additionally, chronic respiratory diseases, such as COPD, can lead to different comorbidities and physical deconditioning, decreasing the health-related quality of life (HRQoL) of patients suffering from these diseases (6).

Oral health is often reported to be affected by respiratory diseases in respective patients; a recent meta-analysis showed that patients with asthma as well as COPD showed an association to the presence of periodontal diseases (7). Besides a direct association, medications, e.g., asthma medication including bronchodilators, corticosteroids, or anticholinergic drugs inhaled by the patients can increase the risk of dry mouth, dental caries, dental erosion, periodontal diseases, and oral candidiasis (8, 9). Especially for severely diseased patients, such as patients with cystic fibrosis or COPD, oral bacteria related to dental and periodontal diseases can also colonize the lungs, increasing the risk for complications (10, 11). Additionally, patients with obstructive sleep apnea, which is completely different from the other respiratory diseases described here, show affected oral health conditions as well as the relationship between oral diseases and the underlying disorder; this is heterogeneous, but obvious for both the children and adults (12). In the complex relationship between oral and respiratory health, the multifactorial character of oral diseases, as well as the anatomic proximity of the oropharyngeal and respiratory tract could be relevant.

A particular issue of interest in this context is the oral HRQoL (OHRQoL). As a part of the general HRQoL, the OHRQoL reflects the perceived affection of HRQoL by conditions related to the oral cavity including teeth, mouth, or dentures (13, 14). In other groups of systemically diseased patients, the OHRQoL showed interesting and clinically relevant results. For example, patients with rheumatic diseases show an impaired OHRQoL, whereby rheumatic disease-related parameters might be major influential factors (15). On the other hand, patients after solid organ transplantation were supposed to undergo a “response shift” of the perception of OHRQoL, i.e., reduced awareness of their insufficient oral health status (16). Considering the impaired oral health, reduced general HRQoL alongside the anatomic proximity between the oral cavity and the respiratory system, the OHRQoL of patients with different respiratory diseases appears a topic of clinical interest. Therefore, this study evaluates the OHRQoL of patients with chronic respiratory diseases. It was hypothesized that these patients would show a reduced OHRQoL.

## Methods

This study was performed in full accordance with the Preferred Reporting Items for Systematic Reviews and Meta-Analyses (PRISMA) (17).

### Patients, Intervention, Comparison, and Outcome (PICO) Question

The PICO question was as follows: “Do patients with severe respiratory diseases show a reduced OHRQoL?”. Thereby, “patients” were individuals with chronic respiratory diseases. The aspect “intervention” was not defined because it was expected to primarily include cross-sectional studies. “Comparison” was a healthy control group, if applicable. Otherwise, values should be compared to reference values or other groups of patients with systemic diseases. Finally, “outcome” was any applied OHRQoL measurement. The hypothesis was formed that the OHRQoL of patients with chronic respiratory diseases would be worse than in healthy individuals.

### Eligibility Criteria

For inclusion in this study, several inclusion criteria were formulated:

- Publication until 30th of June, 2021.
- Examination of children or adults with a chronic respiratory disease or condition.
- Reporting of any OHRQoL measurement.
- Full text in English language.

### Search Strategy

In July 2021, a systematic literature search was performed by two different and independent reviewers. As databases for literature search, the PubMed, Medline, Web of Science, and Scopus were chosen, using the search terms: “oral health-related quality of life” and respiratory disease or lung and “oral health-related quality of life.” A manual search based on the references of findings complemented the systematic literature search. All the findings were checked and screened for their eligibility.

### Data Extraction

After all the articles were screened and checked, qualitative data extraction was applied. Thereby, different issues were extracted from the respective articles.

- Form of respiratory disease, year of publication, number of participants, study type, age, gender, and smoking.
- Presence and characteristics of a healthy control group.
- Oral health findings, if applicable.
- Oral HRQoL assessment including the form of measurement and results.
- Potential relationship between OHRQoL and general parameters, disease-related parameters, or oral health findings, if applicable.
- Subscales of the OHRQoL measurements, if applicable.

Two independent reviewers executed the whole process of systematic search and study selection as well as qualitative analysis. Only studies explicitly reporting OHRQoL of patients with chronic respiratory diseases were considered within this study.

### Quality Assessment

The 11-item checklist from the Agency for Healthcare Research and Quality (AHRQ) for cross-sectional studies was applied for quality assessment of the included studies (18). The answers “no” or “unclear” were rated as 0 and the answer “yes” was rated as 1 point for each question to estimate a score for the respective quality of the studies. A sum score of all the questions of 0–3 indicated low quality, 4–7 indicated moderate quality, and a score of 8–11 indicated high quality of this study. The quality appraisal was conducted by two independent reviewers, whereby any disagreements were discussed and resolved in the whole author group.

## Results

### Search Findings

**Figure 1:**
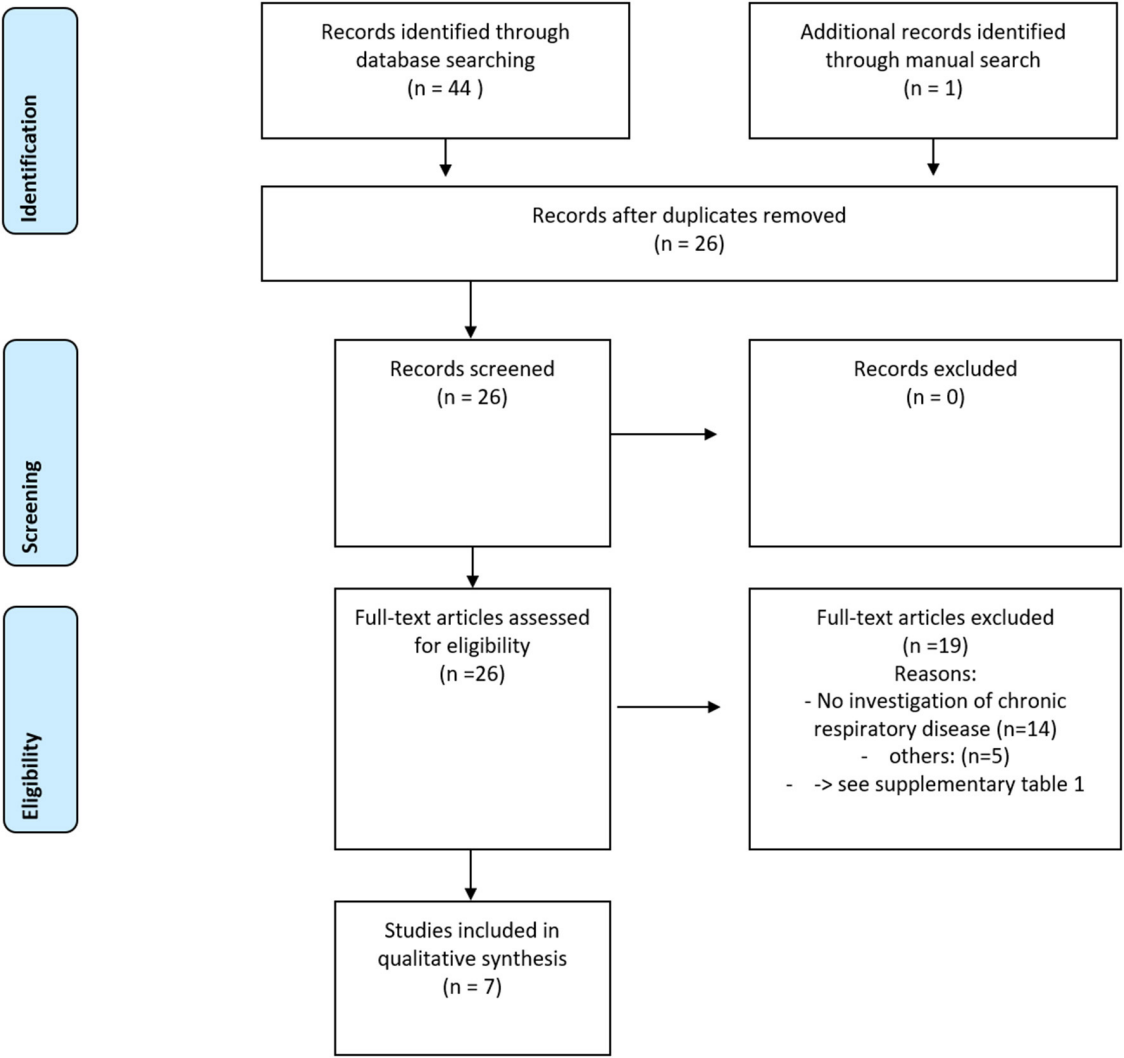
The Preferred Reporting Items for Systematic Reviews and Meta-Analyses (PRISMA) diagram for the systematic review process in this study (17).

The PRISMA diagram, reflecting the findings of the systematic search, is given in Figure 1. Out of 44 database search findings, 26 full-text articles were assessed for their eligibility. During this, 19 articles were excluded (Supplementary Table 1). Finally, seven studies were included in the qualitative analysis.

### Characteristics of the Included Studies

Out of the seven studies, two studies were on patients with COPD (19, 20), while each one study was on cystic fibrosis (21), lung transplantation (22), sleep-disordered breathing (23), obstructive sleep apnea (24), and severe asthma (25). Three studies were performed in children (21, 23, 24), while four studies were conducted in adult patients (19, 20, 22, 25). All the included studies had either a cross-sectional or an observational design. The healthy control group was examined in five of the included studies (20, 22–25). A full overview of the study characteristics is shown in Table 1.

**Table 1:** Overview of the included studies.

**Author, year**	**Disease**	**Country**	**No. of patients**	**Study type**	**Subjects mean age in years**	**Smoking**	**Male (%)**	**Healthy control group for OHRQoL**
Saltness et al. (19)	COPD	Norway	100	Monocentric cross-sectional study	65.9 ± 10.1	39%	56%	No
Patrick et al. (21)	Cystic fibrosis	USA	39	Multicentric cross-sectional	43.6% 8–12 years, 56.4% 13–17 years	n/a	53%	No
Schmalz et al. (22)	Lung transplantation	Germany	60	Monocentric cross-sectional study	54.03 ± 9.97	0%	50%	Yes: *n* = 70, age: 55.44 ± 8.54 years, 37% male
Gaeckle et al. (20)	COPD	USA	20	Monocentric prospective observational study (follow-up: 60 days)	60 (56–68)	50%	60%	Yes: *n* = 10, age: 54.5 (50-60) years, 60% male
Grillo et al. (23)	Sleep-disordered breathing	Italy	61	Monocentric cross-sectional study	12.4 ± 3.1	16.4%	54.1%	Yes: *n* = 61, age: 11.9 ± 2.8 years, 50.8% male
Tamsas et al. (24)	Obstructive sleep apnoea	USA	31	Monocentric cross-sectional study	12.8 ± 3.1	14%	52%	Yes: *n* = 36, age: 11.8 ± 2.2 years, 55% male
Brasil-Oliveira et al. (25)	Severe asthma	Brazil	40	Monocentric cross-sectional study	51.8 ± 10.8	0%	15%	Yes: *n* = 50, age: 48.2 ± 12.4 years, 42% male

*Values are presented as the mean values ± SD, mean values (range), or percentages*.

*OHRQoL, oral health-related quality of life; n/a, not applicable; COPD, chronic obstructive pulmonary disease*.

### Quality Assessment

Quality appraisal revealed that three studies were of high quality (19, 20, 23), while four studies were elevated with moderate quality (21, 22, 24, 25) according to the AHRQ criteria for cross-sectional studies (18) (Table 2).

**Table 2:** Quality assessment of the included studies according to the Agency for Healthcare Research and Quality (ARHQ) (18).

**Item**	**Saltnes et al. (19)**	**Patrick et al. (21)**	**Schmalz et al. (22)**	**Gaeckle et al. (20)**	**Grillo et al. (23)**	**Tamasas et al. (24)**	**Brasil-Oliveira et al. (25)**
(1) Define the source of information (survey, record review)	Yes	Yes	Yes	Yes	Yes	Yes	Yes
(2) List inclusion and exclusion criteria for exposed and unexposed subjects (cases and controls) or refer to previous publications	Yes	No	Yes	Yes	Yes	Yes	Yes
(3) Indicate time period used for identifying patients	Yes	No	Yes	Yes	Yes	No	Yes
(4) Indicate whether or not subjects were consecutive if not population-based	Yes	Yes	Yes	Yes	Yes	Yes	Yes
(5) Indicate if evaluators of subjective components of study were masked to other aspects of the status of the participants	No	No	No	No	No	No	No
(6) Describe any assessments undertaken for quality assurance purposes (e.g., test/retest of primary outcome measurements)	Yes	NA	Yes	Yes	Yes	NA	Yes
(7) Explain any patient exclusions from analysis	Yes	NA	NA	Yes	Yes	NA	NA
(8) Describe how confounding was assessed and/or controlled.	Yes	Yes	U	Yes	Yes	Yes	Yes
(9) If applicable, explain how missing data were handled in the analysis	NA	Yes	NA	NA	NA	NA	NA
(10) Summarize patient response rates and completeness of data collection	Yes	Yes	Yes	Yes	Yes	Yes	Yes
(11) Clarify what follow-up, if any, was expected and the percentage of patients for which incomplete data or follow-up was obtained	NA	NA	NA	Yes	NA	NA	NA
Total score	8	5	6	9	8	5	7

### Oral Health Records and Findings

Reporting of oral health data was not very comprehensive in the majority of included studies. One study did not report on any oral health conditions of the participants (21). The four studies including adults reported on remaining teeth (19, 20, 22, 25). Moreover, four studies reported on dental as well as periodontal conditions, whereby the assessment method was quite different (22–25). The detailed oral health findings, if applicable, are shown in Table 3.

**Table 3:** Oral health parameters and respective main results if they presented as the mean values ± SD, means (range), or percentages in the included studies.

**References**	**Tooth loss, remaining teeth, dentures**	**Dental diseases, caries, dental treatment need**	**Oral hygiene indices**	**Periodontal parameters, periodontal treatment need**	**Further oral health parameters**
Saltness et al. (19)	44% <20 teeth	n/a	n/a	n/a	9% hyposalivation, 39% oral health problems
Patrick et al. (21)	n/a	n/a	n/a	n/a	n/a
Schmalz et al. (22)	M-T: 8.17 ± 5.82^*^	DMF-T: 20.53 ± 5.09, D-T: 0.82 ± 1.85, F-T 11.55 ± 4.57	n/a	98% moderate to severe periodontitis	n/a
Gaeckle et al. (20)	Number of teeth: 16.5 (8.5–23.5)	n/a	PI: 2.2 (1.5–2.8)	n/a	n/a
Grillo et al. (23)	n/a	DMFS: 13.6 ± 4.7, dmfs: 8.5 ± 2.3	n/a	PPD: 2.4 ± 0.5, BOP: 0.9 ± 0.2	n/a
Tamsas et al. (24)	n/a	dmfs: 5.1 ± 8.5, DMFS: 15.2 ± 11.8	n/a	BOP: 87%, PPD mean 2.7 ± 1.3	Comprehensive information on oropharyngeal morphology reported
Brasil-Oliveira et al. (25)	M-T: 7.9 ± 7.2,	D-T: 1.4 ± 2.0, F-T: 4.2 ± 3.7, DMF-T: 13.5 ± 6.5	n/a	Periodontitis: 92.5%	Reduced salivary flow: 80%

*M-T, missing teeth; D-T, decayed teeth; F-T, filled teeth; DMF-T, decayed-, missing-, and filled teeth index; PI, plaque index; GI, gingival index; PPD, periodontal probing depth; n/a, not applicable;*

* *inclusion criterion: at least 6 remaining teeth*.

### Oral Health-Related Quality of Life Measurements and Results

**Figure 2:**
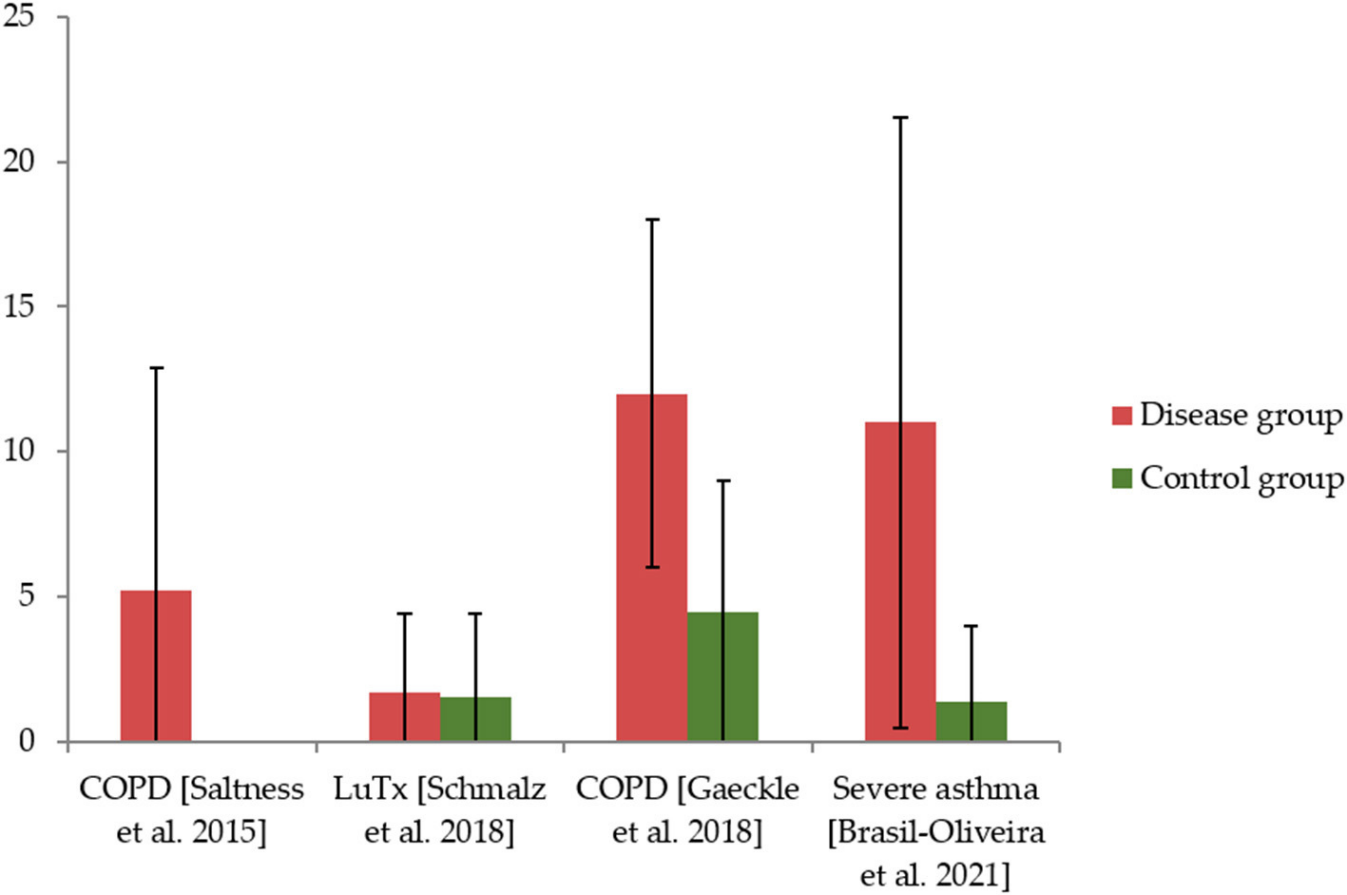
The Oral Health Impact Profile (OHIP) values of included studies on adults and values for healthy controls, if applicable.

**Figure 3:**
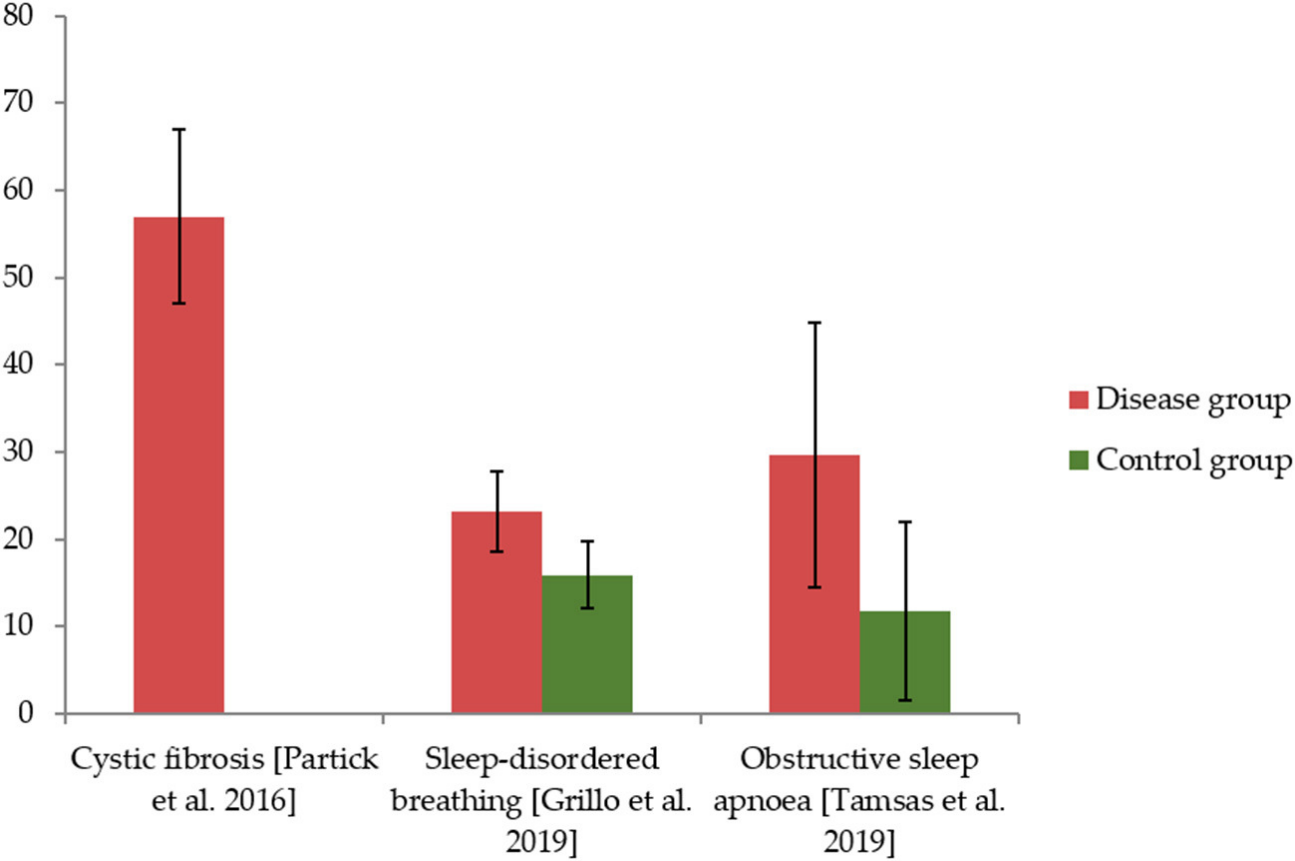
The Child OHIP (COHIP) values for children in included studies and of healthy controls, if applicable.

The four adult studies applied the Oral Health Impact Profile-14 (OHIP-14) for OHRQoL assessment, showing mean values between 1.7 and 12 points in sum scale (Figure 2) (19, 20, 22, 25). The three other studies applied the Child OHIP (COHIP), reporting average sum scores between 23.2 and 67.5 points (Figure 3) (21, 23, 24). Four studies (each two in children and adults) reported a worse OHRQoL in the respiratory disease compared to the healthy control group (20, 23–25), while only one study an adult lung transplant recipients did not show a difference against a healthy control (22). Associations between OHRQoL and HRQoL, disease-related parameters, or oral health conditions were rarely examined and reported, respectively (Table 4). Only one study examining adults (25) and two studies examining children (21, 24) reported on subscales of the OHIP-14 or the COHIP, respectively (Table 5).

**Table 4:** Applied assessments for OHRQoL and relevant results for the included studies.

**References**	**Assessment of OHRQoL**	**OHRQoL worse than healthy control (HC)**	**Association/correlation between OHRQoL and general HRQoL**	**Association/correlation between OHRQoL and oral health**	**Association and/or correlation between OHRQoL and disease-related parameters**
Saltness et al. (19)	OHIP 14: 5.2 ± 7.7	n/a	Higher OHIP 14 related to worse MCS SF-36	Oral health problems related to poorer OHRQoL	n/a
Patrick et al. (21)	COHIP: A: 57.0 ± 10.0, B: 67.5 ± 14.2	n/a	n/a	n/a	Number of medications correlated with better COHIP
Schmalz et al. (22)	OHIP 14: 1.70 ± 2.70	No, OHIP 14: 1.54 ± 2.86	n/a	No associations detected	No
Gaeckle et al. (20)	OHIP 14: 12 (6–18.5)	Yes, OHIP 14: 4.5 (0–8)	n/a	n/a	No
Grillo et al. (23)	COHIP: 23.2 ± 4.6	Yes, COHIP: 15.9 ± 3.8	n/a	Malocclusion	Mallampati class and Obesity correlated with worse COHIP
Tamsas et al. (24)	COHIP: 29.7 ± 15.2	Yes, COHIP: 11.8 ± 10.2	n/a	n/a	n/a
Brasil-Oliveira et al. (25)	OHIP 14: 11.0 ± 10.5 (mild-to-moderate asthma: 6.2 ± 7.4)	Yes, OHIP 14: 1.4 ± 2.6	Higher OHIP 14 correlation with better PCS and MCS of SF-36	n/a	n/a

*OHRQoL, oral health-related quality of life; n/a, not applicable; OHIP, Oral Health Impact Profile; COHIP, Child OHIP; PCS, physical compound summary; MCS, mental compound summary; SF-36, Short Form 36 Health Survey Questionnaire*.

**Table 5:** Subscales of OHRQoL in the included studies, if applicable.

**OHIP 14**							
**References, disease**	**Functional limitation**	**Physical pain**	**Psycho-social discomfort**	**Physical disability**	**Psycho-logical disability**	**Social disability**	**Handicap**
Brasil-Oliveira et al. (25)	1.2 ± 2.0^*^	3.0 ± 2.4^*^	1.8 ± 2.5^*^	2.1 ± 2.3^*^	2.6 ± 2.3^*^	1.1 ± 0.8	0.6 ± 1.6
**OHIP 14**							
**References, disease**	**Oral hgealth well-being**	**Functional well being**	**Social-emotional well-being**	**School environment**	**Self-image**	**Global health**	
Patrick et al. (21)^**^	A: 19.3 ± 4.3, B: 21.9 ± 6.4	A: 7.7 ± 1.4, B: 8.2 ± 3.2	A: 10.1 ± 3.1, B: 13.4 ± 5.2^*^	A: 4.5 ± 0.9, B: 4.9 ± 2.2	A: 12.3 ± 3.9, B: 14.8 ± 2.8^*^	A: 3.8 ± 1.6, B: 4.6 ± 1.4	
Tamasas et al. (24)	14.1 ± 5.5^*^	3.7 ± 3.0^*^	3.5 ± 5.6^*^	1.0 ± 1.7	7.3 ± 5.5^*^	n/a	

*The results are given as the mean values ± SD or otherwise as percentages. OHIP, Oral Health Impact Profile*.

* *significant different from control*.

** *Group A: 8–12 years (n = 17); group B: 13–17 years (n = 22)*.

## Discussion

This study hypothesized that patients with chronic respiratory diseases would show a reduced OHRQoL. Based on the findings of the literature search, this hypothesis can be confirmed, but several disease-specific and methodological issues need further discussion. Thereby, the following will provide: (I) an interpretation of the reduced OHRQoL in patients with chronic respiratory diseases in general and with respect to the different diseases, (II) a discussion of the applied methods alongside with (III) recommendations for further study in this field to strengthen the body of evidence and strive some clinical implications.

(I) Altogether, the OHRQoL of patients suffering from systemic diseases can be discussed controversially because several heterogeneous phenomena, depending on the form of the disease and/or therapy, exist. It is known that oral health can affect general HRQoL (26) and that reduced HRQoL can negatively affect OHRQoL (13, 27). Patients with severe chronic diseases or conditions, e.g., rheumatic diseases or hemodialysis show a reduced OHRQoL caused by their general disease burden including pain and impact on their daily life (15, 28). Thereby, the OHIP-14 values for patients with respiratory diseases in this study were similar as for rheumatic diseased patients or individuals undergoing renal replacement therapy (15, 28). Thus, the HRQoL impairment due to the general disease could be one reason for the reduced OHRQoL in this study; especially, severely diseased individuals with proceeded COPD, cystic fibrosis, and severe asthma are impaired in their everyday life and show affected HRQoL (29–31). Thereby, both severe symptoms, such as breathlessness, as well as mental health issues, such as anxiety or depression, can affect HRQoL outcomes (6, 31), potentially affecting OHRQoL of patients. A special position within this study is obstructive sleep apnea or sleep-disordered breathing, respectively. Although this is not an exclusive respiratory disease, it has been included in this study because it is a chronic condition affecting the respiratory system and is of high relevance for the dentist because oral health issues are common in these individuals (12). These sleep disorders lead to complex suffering of the patients, with an impairment of quality of life in both children and adults (12, 32). Furthermore, oral appliances for therapy or wearing an oxygen mask overnight could affect OHRQoL of respective individuals. Only one patient group, i.e., patients after lung transplantation, what rather represents a therapy than a respiratory disease, showed unaffected OHRQoL (22). It is known that patients after organ transplantation show a response shift, whereby oral health issues are pushed into the background because of the general disease burden (16, 33). While this is the reason for the unaffected OHRQoL of patients after lung transplantation, the other groups did not show this response shift phenomenon. A reasonable explanation for that appears the anatomic proximity between the airways and the oral cavity. Moreover, the association between oral diseases and the included respiratory conditions and/or the respective medication (7–12) could explain the affected OHRQoL. Altogether, the reduced OHRQoL of patients with chronic respiratory diseases appears expedient, but complex. However, the reporting of oral health conditions and HRQoL in the included studies was inconsistent. However, the association between COPD (19) and severe asthma (25) with HRQoL supports the upper mentioned interpretation. The affected OHRQoL of patients with chronic respiratory diseases appears of clinical significance because appropriate management of oral conditions of these systemically ill patients will be needed to positively affect their quality of life. Therefore, sufficient multidisciplinary dental care concepts might be needed, as already demanded for other groups of at-risk patients (15, 16, 28). Until now, there is no specific approach available for a respective dental care concept. It is known that a simple allocation to the dentist does not significantly decrease the dental treatment need of patients with severe general diseases (34). Moreover, respective patients need to receive psychosocial support and sensibilization within a multidisciplinary care concept (16). Therefore, it appears of high importance to apply an individualized, prevention-oriented, and patient-centered dental care concept, focusing on the risk and needs of respective patients (35).

(II) The included studies applied two different methods for OHRQoL assessment, according to the age of included participants. For adult patients, the short form of the OHIP-14 was chosen, which is a validated questionnaire-based tool (36, 37). This questionnaire allows assessing the OHRQoL by 14 different questions, which can be answered on a five-point scale between 0 and 4, where higher values indicate worse OHRQoL. As a patient-reported outcome, the OHRQoL is part of the evidence-based dentistry, and applying the OHIP-14 allows conclusions on the impairment of the domains, such as oral function, psychosocial impact, oral pain, and orofacial appearance (14, 38). Although the OHIP-14 is a validated and commonly used instrument, it is not specific for generally diseased individuals, what potentially limits this method in that case (15, 16, 28). The other measurement, which was applied for children, was the COHIP. This instrument is also validated and was found to present reliable results by the assessment of 34 items and five conceptually distinct subscales: oral health, functional well-being, social/emotional well-being, school environment, and self-image (39). Similar as for the OHIP-14, higher values represent a worse OHRQoL. Thereby, the assessment of the OHRQoL of children is difficult because children often have difficulties to express their concerns in clinical environments and they are largely influenced by socioenvironmental factors of their family and caregivers (40, 41). Although the COHIP findings perceived by the caregivers are sometimes reported, this was not considered in this study because it was aimed to exclusively display the perspective of patient. Within the included studies, the COHIP values between children and parents did not differ in a clinically relevant manner, so it seems reasonable to omit this issue in this study (23, 24). Accordingly, the applied OHRQoL measurement appears reasonable in the included studies, but the major flaw appears the rarely applied investigation on the relationship between OHRQoL and HRQoL, disease-related parameters, and, particularly, oral health.

(III) Some recommendations can be provided for future study in the field. The comprehensive assessment of oral health parameters and their consideration as an influential factor in patients with respiratory diseases would be helpful to evaluate this issue. Additionally, assessment of HRQoL alongside with disease-specific parameters as well as mental problems or conditions would help to gain a deeper understanding. Thereby, the different dimensions/subcategories of OHRQoL measurement should be addressed. Multicenter, prospective study designs, especially considering any dental or medical intervention, would also bring benefit to the understanding of OHRQoL and possibilities to its improvement in patients with respiratory diseases. Reference values and minimal important differences to interpret the clinical relevance of the data would also be of research interest in this study. Altogether, the body of evidence with respect to OHRQoL of patients with chronic respiratory diseases is quite weak, making more study in the field recommendable.

## Strengths and Limitations

This is the first systematic study in this field. The methodology was sound, according to the PRISMA guidelines, and included a quality appraisal of the included studies. This quality appraisal revealed that the included studies were of moderate-to-high quality. The main points of criticism were missing blinding of examination and no consideration of any follow-up in most studies. Altogether, the included studies can be seen as of appropriate quality because the most relevant issues of quality appraisal were addressed by those investigations. The inclusion of such a heterogeneous combination of diseases and of children and adults together limits the comparability of findings. On one hand, the different diseases can cause different intra- and extraoral effects, potentially affecting OHRQoL of patients. For example, COPD and asthma are associated with periodontitis (7), while other included respiratory diseases are not associated with periodontitis. Periodontitis leads to reduced OHRQoL (42), what might be of relevance in the respective diseases, which are related to periodontal conditions. As this is just one potential example for the heterogeneity of the included diseases, the comparability of respective studies in this study is very limited. However, this study aimed to gain insight into the OHRQoL of patients with chronic respiratory diseases and not directly to compare the different diseases to each other. Similarly, the rationale for including children and adults together can be seen critically. Thereby, a direct comparison between the disease groups was not possible; but, due to the low number of studies on OHRQoL of the respective patient groups, it was decided to include studies on children, too. Of course, it is not possible to compare adults and children suffering from different respiratory diseases. To allow a comprehensive view on the OHRQoL of patients with chronic respiratory diseases, the inclusion appears reasonable, irrespective of the heterogeneity. Only seven studies were considered within this study, limiting the ability to draw meaningful conclusion. Moreover, the analysis was just qualitative. The low number of included studies is an important limitation, but is also an important result. The search terms were quite broad and a very comprehensive manual literature search was applied, checking the reference lists of all the included studies. Thereby, no additional findings could be detected. Therefore, this field of study appears understudied, yet, requiring an increased audience in the future. For this, this study provided several recommendations for future study in the field. More study will be necessary to gain insight into the OHRQoL of patients with respiratory diseases; this study can provide a basis for future study in the field.

## Conclusion

Patients with chronic respiratory diseases show a reduced OHRQoL. This could be caused by a higher prevalence of oral diseases and underlying disease burden that need further clarification in future studies. An interdisciplinary dental care to support oral health and OHRQoL could be recommendable in individuals with chronic respiratory diseases.

## Data Availability Statement

The original contributions presented in the study are included in the article/Supplementary Material, further inquiries can be directed to the corresponding authors.

## Author Contributions

SL, WN, and WW conceptualized the research, conducted a systematic review, analyzed and interpreted the results, and wrote the manuscript. DZ and AA participated in data analysis and interpretation and revised the manuscript. GS, JZ, SH, and HX administrated and supervised the whole research project. All the authors have read and approved the final version of the manuscript.

## Funding

This work was funded by Science Research Cultivation Program of Stomatological Hospital, Southern Medical University [No. PY2020004 for SL (simin.li.dentist@gmail.com) and No.PY2021002 for WN (wanchenning0627@gmail.com)].

## Conflict of Interest

The authors declare that the research was conducted in the absence of any commercial or financial relationships that could be construed as a potential conflict of interest.

## Publisher's Note

All claims expressed in this article are solely those of the authors and do not necessarily represent those of their affiliated organizations, or those of the publisher, the editors and the reviewers. Any product that may be evaluated in this article, or claim that may be made by its manufacturer, is not guaranteed or endorsed by the publisher.
