# Supplementary material for: Oral Health-Related Quality of Life in Patients With Chronic Respiratory Diseases—Results of a Systematic Review
Source: Front Med (Lausanne). 2022 Jan 12;8:757739. doi: 10.3389/fmed.2021.757739 (PMC8790480; doi:10.3389/fmed.2021.757739)
Supplement: Supplementary file 1 [file Table_1.DOCX]

**Supplementary material**

**Supplementary Table 1**: Excluded full-text articles screened for eligibility with reason for exclusion.

| **Author and year** | **Reason for exclusion** |
| --- | --- |
| *Fischer et al. 2020* | Review |
| *Schmalz et al. 2021* | Review |
| *Schmalz et al. 2021* | not explicitely LUTx reported |
| *Knorst et al. 2021* | no chronic respiratory disease |
| *Benson et al. 2010* | no chronic respiratory disease |
| *Emami et al. 2014* | study protocol |
| *Lee et al. 2020* | no chronic respiratory disease |
| *Pereira et al. 2009* | no chronic respiratory disease |
| *Puturdze et al. 2019* | no chronic respiratory disease and no OHRQoL reporting |
| *Strini et al. 2011* | no chronic respiratory disease |
| *Samuel et al. 2021* | no chronic respiratory disease |
| *Degrimenci et al. 2021* | no chronic respiratory disease |
| *Lopez et al. 2019* | Meta analysisn no chronic respiratory disease |
| *Emami et al. 2012* | no chronic respiratory disease, no OHRQoL outcome |
| *Tsukamoto et al. 2017* | no chronic respiratory disease, no OHRQoL outcome |
| *Eriksen et al. 2019* | no chronic respiratory disease, no OHRQoL outcome |
| *Antunes et al. 2016* | no chronic respiratory disease |
| *Albrecht et al. 2016* | no chronic respiratory disease, Metaanalysis |
| *Barkokebas et al. 2015* | no chronic respiratory disease |

OHRQoL: oral health-related quality of life

**References Supplementary Table 1**

S1. Fischer RG, Lira Junior R, Retamal-Valdes B, Figueiredo LC, Malheiros Z, Stewart B, Feres M. Periodontal disease and its impact on general health in Latin America. Section V: Treatment of periodontitis. Braz Oral Res. 2020 Apr 9;34(supp1 1):e026. doi: 10.1590/1807-3107bor-2020.vol34.0026. PMID: 32294679.

S2. Schmalz G, Garbade J, Kollmar O, Ziebolz D. Does oral health-related quality of life of patients after solid organ transplantation indicate a response shift? Results of a systematic review. BMC Oral Health. 2020 Dec 9;20(1):356. doi: 10.1186/s12903-020-01350-w. PMID: 33298051; PMCID: PMC7726902.

S3. Schmalz G, Garbade J, Sommerwerck U, Kollmar O, Ziebolz D. Oral health-related quality of life of patients after solid organ transplantation is not affected by oral conditions: results of a multicentre cross-sectional study. Med Oral Patol Oral Cir Bucal. 2021 Jul 1;26(4):e437-e444. doi: 10.4317/medoral.24277. PMID: 33340076; PMCID: PMC8254886.

S4. Knorst JK, Brondani B, Tomazoni F, Vargas AW, Cósta MD, da Silva Godois L, Mendes FM, Ardenghi DM, Ardenghi TM. COVID-19 pandemic reduces the negative perception of oral health-related quality of life in adolescents. Qual Life Res. 2021 Jun;30(6):1685-1691. doi: 10.1007/s11136-021-02757-w. Epub 2021 Jan 21. PMID: 33475914; PMCID: PMC7819148.

S5. Benson P, O'Brien C, Marshman Z. Agreement between mothers and children with malocclusion in rating children's oral health-related quality of life. Am J Orthod Dentofacial Orthop. 2010 May;137(5):631-8. doi: 10.1016/j.ajodo.2008.06.033. PMID: 20451782.

S6. Emami E, Nguyen PT, Almeida FR, Feine JS, Karp I, Lavigne G, Huynh N. The effect of nocturnal wear of complete dentures on sleep and oral health related quality of life: study protocol for a randomized controlled trial. Trials. 2014 Sep 13;15:358. doi: 10.1186/1745-6215-15-358. PMID: 25218696; PMCID: PMC4177759.

S7. Lee VHK, Grant CG, Mittermuller BA, Singh S, Weiss B, Edwards JM, Schroth RJ. Association between early childhood oral health impact scale (ECOHIS) scores and pediatric dental surgery wait times. BMC Oral Health. 2020 Oct 17;20(1):285. doi: 10.1186/s12903-020-01263-8. PMID: 33069219; PMCID: PMC7568462.

S8. Pereira TC, Brasolotto AG, Conti PC, Berretin-Felix G. Temporomandibular disorders, voice and oral quality of life in women. J Appl Oral Sci. 2009;17 Suppl(spe):50-6. doi: 10.1590/s1678-77572009000700009. PMID: 21499655; PMCID: PMC5467364.

S9. Puturidze S, Margvelashvili M, Bilder L, Kalandadze M, Margvelashvili V. CORRELATION OF ORAL HEALTH STATUS WITH GENERAL HEALTH IN ELDERLY LIVING AT RESIDENTIAL HOMES IN GEORGIA. Georgian Med News. 2019 Jul-Aug;(292-293):21-25. PMID: 31560657.

S10. Strini PJ, Strini PJ, De Souza Barbosa T, Duarte Gavião MB. Assessment of orofacial dysfunctions, salivary cortisol levels and oral health related quality of life (ORHQoL) in young adults. Arch Oral Biol. 2011 Dec;56(12):1521-7. doi: 10.1016/j.archoralbio.2011.06.009. Epub 2011 Jul 18. PMID: 21763639.

S11. Samuel SR, Kuduruthullah S, Khair AMB, Shayeb MA, Elkaseh A, Varma SR. Dental pain, parental SARS-CoV-2 fear and distress on quality of life of 2 to 6 year-old children during COVID-19. Int J Paediatr Dent. 2021 May;31(3):436-441. doi: 10.1111/ipd.12757. Epub 2021 Jan 25. PMID: 33220088; PMCID: PMC7753551.

S 12. Degirmenci K, Kalaycioglu O. Evaluation of quality of life and oral hygiene attitudes of individuals using dental prostheses during the COVID-19 pandemic. J Prosthet Dent. 2021 Jul;126(1):51.e1-51.e7. doi: 10.1016/j.prosdent.2021.03.022. Epub 2021 May 24. PMID: 34034897; PMCID: PMC8141906.

S13. Lopez D, Waidyatillake N, Zaror C, Mariño R. Impact of uncomplicated traumatic dental injuries on the quality of life of children and adolescents: a systematic review and meta-analysis. BMC Oral Health. 2019 Oct 22;19(1):224. doi: 10.1186/s12903-019-0916-0. PMID: 31640671; PMCID: PMC6805369.

S14. Emami E, Lavigne G, de Grandmont P, Rompré PH, Feine JS. Perceived sleep quality among edentulous elders. Gerodontology. 2012 Jun;29(2):e128-34. doi: 10.1111/j.1741-2358.2010.00426.x. Epub 2010 Oct 4. PMID: 21029154.

S15. Tsukamoto M, Hitosugi T, Esaki K, Yokoyama T. The Anesthetic Management for a Patient With Trisomy 13. Anesth Prog. 2017 Fall;64(3):162-164. doi: 10.2344/anpr-64-02-09. PMID: 28858551; PMCID: PMC5579817.

S16. Schilbred Eriksen E, Gulati S, Moen K, Wisth PJ, Løes S. Apnea-Hypopnea Index in Healthy Class III Patients Treated With Intraoral Vertical Ramus Osteotomy: A Prospective Cohort Study. J Oral Maxillofac Surg. 2019 Mar;77(3):582-590. doi: 10.1016/j.joms.2018.07.017. Epub 2018 Jul 24. PMID: 30118665.

S17. Antunes LA, Castilho T, Marinho M, Fraga RS, Antunes LS. Childhood bruxism: Related factors and impact on oral health-related quality of life. Spec Care Dentist. 2016 Jan;36(1):7-12. doi: 10.1111/scd.12140. Epub 2015 Sep 21. PMID: 26388123.

S18. Albrecht M, Kupfer R, Reissmann DR, Mühlhauser I, Köpke S. Oral health educational interventions for nursing home staff and residents. Cochrane Database Syst Rev. 2016 Sep 30;9(9):CD010535. doi: 10.1002/14651858.CD010535.pub2. PMID: 27689868; PMCID: PMC6457754.

S19. Barkokebas A, Silva IH, de Andrade SC, Carvalho AA, Gueiros LA, Paiva SM, Leão JC. Impact of oral mucositis on oral-health-related quality of life of patients diagnosed with cancer. J Oral Pathol Med. 2015 Oct;44(9):746-51. doi: 10.1111/jop.12282. Epub 2014 Oct 25. PMID: 25345344.
